# Cdx2 Regulates Intestinal EphrinB1 through the Notch Pathway

**DOI:** 10.3390/genes12020188

**Published:** 2021-01-28

**Authors:** Yalun Zhu, Alexa Hryniuk, Tanya Foley, Bradley Hess, David Lohnes

**Affiliations:** 1 Department of Cellular and Molecular Medicine, University of Ottawa, 451 Smyth Road, Ottawa, ON K1H 8M5, Canada; yzhu146@uottawa.ca (Y.Z.); Alexa.Hryniuk@umanitoba.ca (A.H.); tfole066@uottawa.ca (T.F.); bhess@uottawa.ca (B.H.); 2 Department of Human Anatomy and Cell Science, Rady Faculty of Health Sciences, Max Rady College of Medicine, University of Manitoba, 745 Bannatyne Avenue, Winnipeg, MB R3E 0J9, Canada

**Keywords:** Cdx, Notch, EphrinB1, differentiation, SW480, colorectal cancer

## Abstract

The majority of colorectal cancers harbor loss-of-function mutations in *APC*, a negative regulator of canonical Wnt signaling, leading to intestinal polyps that are predisposed to malignant progression. Comparable murine *APC* alleles also evoke intestinal polyps, which are typically confined to the small intestine and proximal colon, but do not progress to carcinoma in the absence of additional mutations. The Cdx transcription factors Cdx1 and Cdx2 are essential for homeostasis of the intestinal epithelium, and loss of Cdx2 has been associated with more aggressive subtypes of colorectal cancer in the human population. Consistent with this, concomitant loss of Cdx1 and Cdx2 in a murine *APC* mutant background leads to an increase in polyps throughout the intestinal tract. These polyps also exhibit a villous phenotype associated with the loss of EphrinB1. However, the basis for these outcomes is poorly understood. To further explore this, we modeled Cdx2 loss in SW480 colorectal cancer cells. We found that Cdx2 impacted Notch signaling in SW480 cells, and that *EphrinB1* is a Notch target gene. As EphrinB1 loss also leads to a villus tumor phenotype, these findings evoke a mechanism by which *Cdx2* impacts colorectal cancer via Notch-dependent EphrinB1 signaling.

## 1. Introduction

In mice, intestinal epithelial cells are replenished every five to seven days by intestinal stem cells resident near the base of the crypt [1,2]. These stem cells give rise to rapidly dividing transit-amplifying cells that subsequently differentiate into the mature cells of the intestinal epithelium, comprised of absorptive (enterocytes) and secretory (Goblet, Paneth and enteroendocrine) lineages [3].

Colorectal cancer (CRC) is the third leading cause of cancer-related mortality worldwide [4]. The predominant initial genetic lesions underlying CRC are inactivating mutations of the *adenomatous polyposis coli* (*APC*) gene, which encodes a negative regulator of the canonical Wnt pathway. Such mutations cause an increase in Wnt signalling, leading to hyperproliferation of *APC* mutant intestinal cells and formation of adenomatous polyps [5,6,7]. Accumulation of subsequent mutations, such as in *KRAS*, as well as tumor-specific genomic imbalances, results in progression of such adenomas to carcinoma [8,9].

Members of the caudal type homeobox (Cdx) family of transcription factors, Cdx1 and Cdx2, are essential for development of the murine intestinal tract and have overlapping roles in homeostasis of the adult intestinal epithelium [10,11,12,13,14,15]. There is considerable evidence suggesting that Cdx2 status also impacts the CRC phenotype. For example, ~30% of human CRC exhibits loss of Cdx2, and this is associated with higher tumor grade [5,6,7]. Furthermore, the frequency of polyps in *APC^Min+/−^* mice, or those induced by azoxymethane, is increased in a *Cdx2* heterozygote background [16,17]. Finally, the loss of Cdx2 is associated with stage II/III CRC patients at high risk of disease progression; such patients have also been shown to benefit from adjuvant chemotherapy, underscoring Cdx2 status as a biomarker [10].

The utility of murine models to explore Cdx function in the intestine has previously been limited by the peri-implantation lethality of *Cdx2* null mouse mutants [18] and potential functional overlap between Cdx1 and Cdx2, which are co-expressed throughout the intestinal epithelium [13]. To address these limitations, a conditional mutagenesis strategy was used to delete *Cdx2* in the intestine in a *Cdx1* null background, and to cross these with the *APC^Min+/−^* model of CRC. Mosaic Cdx loss of function using this approach results in a marked increase in polyposis throughout the intestinal tract. In addition, such *Cdx1:Cdx2:APC^Min^* compound mutants exhibit a highly penetrant villous tumor phenotype coincident with loss of *EphrinB1* expression [19]. Eph-Ephrin signaling plays essential roles in cell sorting processes along the crypt-villus axis, and deletion of *EphrinB1* also evokes villus tumors in an *APC^Min^* background [20]. However, while these findings suggest a molecular basis for the villus tumors in *Cdx2*-*APC^Min^* animals, *EphrinB1* does not appear to be a direct Cdx target gene [19].

In the present study, we further explored the impact of *Cdx* on intestinal tumorigenesis using CRC-derived SW480 cells. Consistent with studies in mice, siRNA-mediated loss of Cdx2 in SW480 cells led to an acceleration of growth and other indices of transformation. In addition, knockdown of Cdx2 resulted in a reduction in *EphrinB1* expression, consistent with prior in vivo observations. Furthermore, Cdx2 loss impacted Notch signaling, both in SW480 cells and in vivo consistent with our prior work [21]. Finally, we present evidence that *EphrinB1* is a direct Notch target gene. These findings suggest a previously unrecognized pathway for Cdx in modulating Eph-Ephrin signaling, and the CRC phenotype, through the Notch signaling pathway.

## 2. Materials and Methods

### 2.1. Generation of Stable Cell Lines

Human colorectal cancer SW480 (ATCC: CCL-228) cells were cultured in Dulbecco’s modified Eagle’s medium supplemented with 10% fetal calf serum (FCS) and 1% penicillin/steptomycin at 37 °C with 5% CO_2_ in air. SW480 cells were transfected with shRNA for Cdx2 or control shRNA vectors (Dharmacon, Denver, CO, USA) using Lipofectamine (Thermofisher, Nepean, ON, Canada) and selected by culture in the presence of 15 μg/mL of puromycin. Surviving clones were isolated and expanded, and Cdx2 expression was assessed by Western blot analysis. *Cdx2* null HEK293 cell lines were generated by CRISPR-Cas9, as described previously [22].

### 2.2. Western Blot Analysis

Cells were disrupted using RIPA lysis buffer and cleared by centrifugation. Proteins were resolved on a 12% SDS-PAGE gel, transferred to a PVDF membrane (Millipore, Etobicoke, ON, Canada), which was blocked with 5% non-fat milk powder in PBS: 0.1% Tween 20 (PBST), then incubated overnight with the appropriate primary antibody at 4°C. Primary antibodies used were rabbit polyclonal anti-Cdx2 (1/1000 dilution, Savory et al., 2009); anti-NICD (1/500 dilution, BD Biosciences); anti-β-actin (1/2000 dilution, Abcam, Branford, CT, USA) or anti-CyclophilinB (1/10,000 dilution, Abcam). Membranes were then washed, incubated with secondary antibodies (HRP-conjugated anti-rabbit or anti-mouse IgG, as appropriate; 1/25,000 dilution, Santa Cruz Biotechnology, Dallas, TX, USA) and immunoreactivity detected by ECL (Millipore) according to the manufacturer’s instructions.

### 2.3. Quantitative Reverse Transcriptase Polymerase Chain Reaction (RT-qPCR)

RNA was extracted from cells or mouse small intestinal epithelial cells with Trizol reagent (Invitrogen, Waltham, MA, USA) and used to generate cDNA by standard procedures. cDNA was subsequently amplified using primers directed against *Cdx2*, *Dll1*, *Dll4*, *EphrinB1*, *Hes1*, *Tff3*, *Math1*, *Lgr5*, *Smoc2* or *β-actin*. qPCR was performed using a MX3005P thermocycler (Agilent Technologies, Mississauga, ON, Canada) and analyzed using the 2^−ΔΔCt^ method [23], normalized to β-actin. Data are from of a minimum of 3 independent biological samples. Error bars are an expression of the mean +/− SD.

### 2.4. Animals

*Cdx1^−/−^*, *Cdx2^f/f^*, and *Villin*-*CreER^T^* mice have been previously described [13,24,25]. To effect *Cdx2* deletion, Cre-positive animals were treated with a single 2 mg dose of tamoxifen by oral gavage; non-transgenic animals, treated in an identical manner, were used as negative controls. Inhibition of Notch signaling was accomplished by treatment with 3 mMol/kg of DAPT (Sigma, St. Louis, MO, USA) by oral gavage for 5 consecutive days. Animals were used at approximately 2 months of age and were maintained according to the guidelines established by the Canadian Council on Animal Care, as approved by the Animal Care & Veterinary Services of the University of Ottawa.

### 2.5. Histology and Immunohistochemistry

Intestines were prepared as previously described [13]. Paraffin-embedded material was sectioned at 5 μm. Immunostaining was carried out using standard methods with an α-Cdx2 antibody at 1/1000 dilution [24] and HRP-conjugated goat α-rabbit secondary antibody (1/1000 dilution, Santa Cruz Biotechnology, Dallas, TX, USA). Slides were mounted using Permount (Fisher) and images captured using a Mirax Midi Scanner (Zeiss, North York, ON, Canada).

### 2.6. In Situ Hybridization

Intestinal sections were cut at 10 μm and slides were processed as specified above. Probes for *Hes1* were synthesized using the DIG RNA labeling system (Roche, Mississauga, ON, Canada), according to the manufacturers recommendations. In situ hybridization was carried out as previously described [26], and slides were mounted using Dako Faramount Aqueous Mounting Medium (Agilent, Santa Clara, CA, USA).

### 2.7. Chromatin Immunoprecipitation (ChIP)-PCR

ChIP was performed as previously described [24], using chromatin generated from SW480 cells. PCR was directed across regions encompassing potential RPBJ or Cdx binding sites, using *Hes1* or *Dll1*, respectively, as positive controls.

### 2.8. Promoter Analysis

pXP2-based luciferase reporter constructs were derived from PCR amplicons of 2kb genomic sequences 5′ of the *EphrinB1* promoter, including putative Notch-effector RBPJ binding sites. RBPJ binding sites were mutagenized using the QuikChange Site-Directed mutagenesis system (Stratagene, La Jolla, CA, USA), according to the manufacturer’s instructions. *Cdx2* and *NICD* expression vectors, and wild type and mutant *Hes1* promoter reporters, have been described previously [27,28]. Transfections were performed in triplicate using jetPRIME (Polyplus) in SW480 cells and lipofectamine in HEK293 cells using 1 μg of the appropriate luciferase reporter construct, 0–500 ng of *NICD* or *Cdx2* expression vectors or empty expression vector, 0.2 μg of *β-galactosidase* expression vector and 100 ng of *GFP* expression vector to a total of 2 μg of DNA per transfection. Cells were harvested 48 h post-transfection, and lysates analyzed using the Promega Luciferase Assay System, according to the manufacturer’s instructions and normalized for transfection efficiency by β-galactosidase activity assessed by the chlorophenol red β-D-galactopyranoside reactivity as previously described [24].

### 2.9. DAPT and Valproic Acid Treatment

SW480 cells and HEK293 cells were treated with either DAPT (0–100 μM) (Sigma) or Valproic acid (VPA) (5 mM) (ICN Biochemicals) and harvested 36 h post-treatment.

### 2.10. Anchorage Independent Growth Assays

SW480 cells were collected and suspended in 2Xmedia with 20% FBS at a density of 1 × 10^5^ cells/mL and plated in 0.3% low melting point agarose on a 0.5% base layer. Media (500 μL) was replenished twice weekly and colonies visualized with a dissecting microscope after 14 days in culture.

## 3. Results

### 3.1. Derivation of CDX2-Deficient SW480 Cells

**Figure 1:**
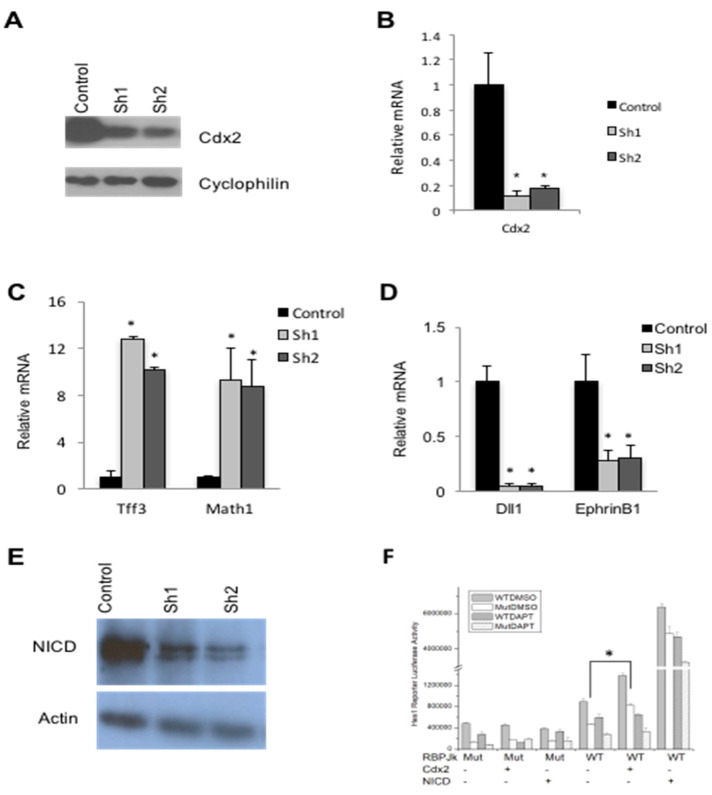
Loss of Cdx2 disrupts Notch signaling. (**A**) Western blot analysis for Cdx2 in control and Sh knockdown cells. CyclophilinB was used as a loading control. (**B**) RT-PCR analysis for expression of *Cdx2* from control compared to Sh knockdowns. (**C**) RT-PCR from SW480 cells for secretory cell markers in control and CDX2 knockdowns. (**D**) Western blot for the Notch Intracellular Domain (NICD) in control and Cdx2 knockdown SW480 cells. Actin was used for a loading control. (**E**) RT-PCR from SW480 cells for *DLL1* and *EPHRINB1* in control and Cdx2 knockdown cultures. (**F**) *Cdx2*, *NICD* or empty expression vectors were transfected into HEK293 cells with *Hes1* wild type or mutated RBPJ binding motif reporter. An amount of 100 μM of DAPT or an equivalent volume of vehicle was added 12 h post-transfection. Error bars represent the standard deviation from 3 independent samples. * *p* < 0.01 by Student’s *t*-test.

The means by which Cdx2 impacts the CRC phenotype is poorly understood. To further explore this, we assessed the consequence of Cdx2 attenuation in CRC-derived SW480 cells. Independent SW480 clonal lines, designated Sh1 and Sh2 were derived and exhibited an ~80–85% reduction in Cdx2 protein and mRNA (Figure 1A,B, respectively). Cdx1, the only other Cdx member expressed in the intestine, was not detected in parental or Cdx2-deficient SW480 cells (data not shown).

### 3.2. Cdx2 Impacts Notch Signaling in SW480 Cells

Cdx2 is necessary for normal intestinal epithelial differentiation in the adult mouse [29]. Consistent with this, Cdx2-deficient SW480 cell lines exhibited an increase in the expression of the secretory cell makers *Tff3* and *Math1* (Figure 1C), indicative of altered differentiation. A similar increase in the levels of secretory cell markers was also seen following disruption of the Notch signaling pathway [30,31]. Coincident with this, expression of the Notch ligand *Dll1* was also attenuated in Cdx2-deficient SW480 cells (Figure 1D), consistent with prior work demonstrating that *Dll1* is a direct Cdx target gene [21]. *EphrinB1* expression was similarly impacted (Figure 1D).

Activation of the Notch pathway results in the proteolytic release of the Notch intracellular domain (NICD) from the membrane that translocates to the nucleus, associates with the transcription factor RBPJ resident at Notch target genes, leading to an increase in their expression [32]. Cdx2-deficient SW480 cells exhibited a decrease in NICD levels compared to controls (Figure 1E). These findings suggest that the decreased *Dll1* level observed in Cdx2 knockdown cells leads to diminution of NICD and subsequent attenuation of Notch signaling. To further examine this relationship, we used a reporter derived from the Notch target *Hes1* [33]. Wild type or *Cdx2* null HEK293 cells were co-transfected with the wild type reporter or one harboring a mutant RBPJ binding motif, with or without *Cdx2* or *NICD* expression vectors (Figure 1F). Cdx2 induced expression from the *Hes1* luciferase reporter, which was blocked by the Notch inhibitor DAPT. *Hes1* luciferase reporter activity was also increased by overexpression of NICD irrespective of DAPT, consistent with DAPT functioning upstream of NICD-dependent transcription [34]. These observations are in agreement with a role of Cdx2 in positively impacting Notch target gene expression.

### 3.3. Cdx2 Regulates EphrinB1 through the Notch Pathway

**Figure 2:**
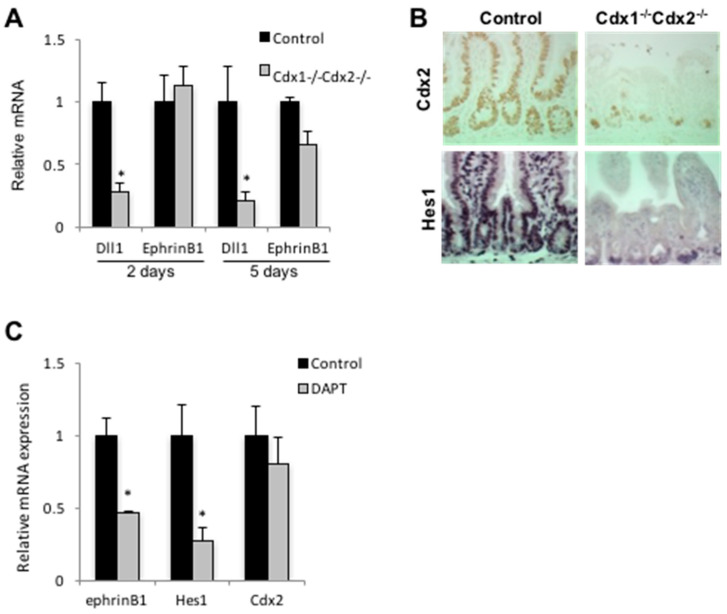
*EphrinB1* and *Dll1* are Cdx-dependent. (**A**) RT-PCR for *Dll1* and *EphrinB1* expression two and five days post-deletion of *Cdx2* from the small intestine of control and *Cdx1^−/−^Cdx2^−/−^* mice. (**B**) Expression of Cdx2 and *Hes1* in the small intestine of control and *Cdx1^−/−^Cdx2^−/−^* mice. (**C**) RT-PCR for *EphrinB1*, *Hes1* and *Cdx2* from the small intestine of control and *Cdx1^−/−^Cdx2^−/−^* treated mice after 5 days of treatment with DAPT. Error bars represent the standard deviation from three independent samples. * *p* < 0.01 by Student’s *t*-test.

*Cdx2* deletion in *APC^min^* mice results in the formation of villous, rather than tubular, polyps, and this outcome is associated with loss of EphrinB1 [19]. Consistent with this, a reduction of *EphrinB1* expression was also seen in Cdx2-deficient SW480 cells (Figure 1D). *EphrinB1* is, however, not impacted by acute *Cdx2* deletion in the intestine, in contrast to expression of the direct Cdx target gene *Dll1* (Figure 2A). In addition, ChIP analysis failed to reveal occupancy by Cdx1 or Cdx2 at the *EphrinB1* locus in murine intestinal epithelial cells (data not shown), suggesting an independent mechanism of regulation.

Prior work has shown that *EphrinB1* is Notch-responsive [35], suggesting that Cdx2 may impact *EphrinB1* through the Notch pathway [36]. Consistent with this, the expression of both *EphrinB1* and the Notch target gene *Hes1* were attenuated in the murine small intestine five days post-*Cdx2*-deletion (Figure 2A,B). To further assess this relationship, we blocked Notch signaling in vivo using the γ-secretase inhibitor DAPT. While this treatment did not perturb *Cdx2* expression, it evoked a decrease in intestinal expression of both *EphrinB1* and *Hes1* to levels similar to those observed following *Cdx2* deletion (Figure 2C).

**Figure 3:**
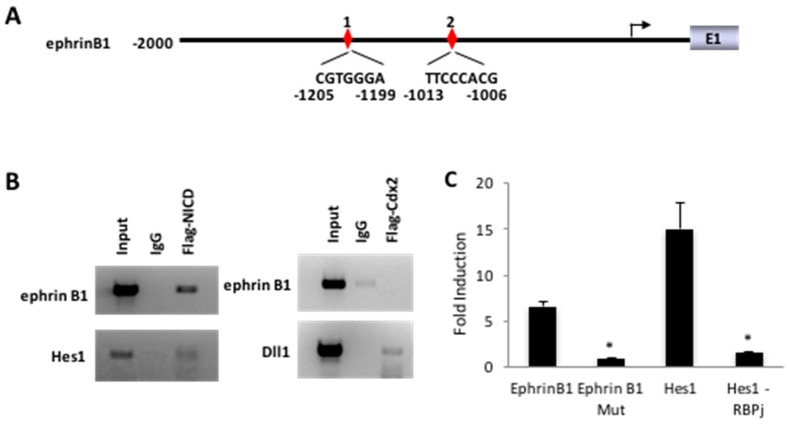
EphrinB1 is a Notch target gene. (**A**) Schematic representation of putative RBPJ binding sites in the *EphrinB1* promoter. (**B**) Chromatin immunoprecipitation (ChIP)-PCR analysis of the *Ephrin B1* locus from SW480 cells showing NICD occupancy (left panel) of the regions encompassing the RBPJ binding sites. Cdx2 did not occupy the *EphrinB1* promoter (right panel). *Hes1* and *Dll1* were used as positive controls for *NICD* and *Cdx2*, respectively. (**C**) Notch signaling induces expression from the *EphrinB1* promoter in cell-based assays in wild type, but not RBPJ binding motif mutant (Mut), reporters. *Hes1* and *Hes1* RBPJ binding motif mutant (Hes1-RPBj) expression vectors were used as positive and negative controls, respectively. Error bars represent the standard deviation from three independent samples. * *p* < 0.05 relative to control by Student’s *t*-test.

The above findings evoke a pathway wherein Cdx2 regulates *EphrinB1* secondary to its impact on *Dll1* and downstream Notch signaling. Consistent with this, the Transcriptional Element Search System (TESS) identified potential RBPJ binding sites in the proximal *EphrinB1* promoter (Figure 3A), while ChIP analysis from SW480 cells revealed that NICD was enriched on this interval in a manner comparable to that observed for the *Hes1* promoter. In contrast, Cdx2 did not appear to associate with the *EphrinB1* promoter (Figure 3B).

**Figure 4:**
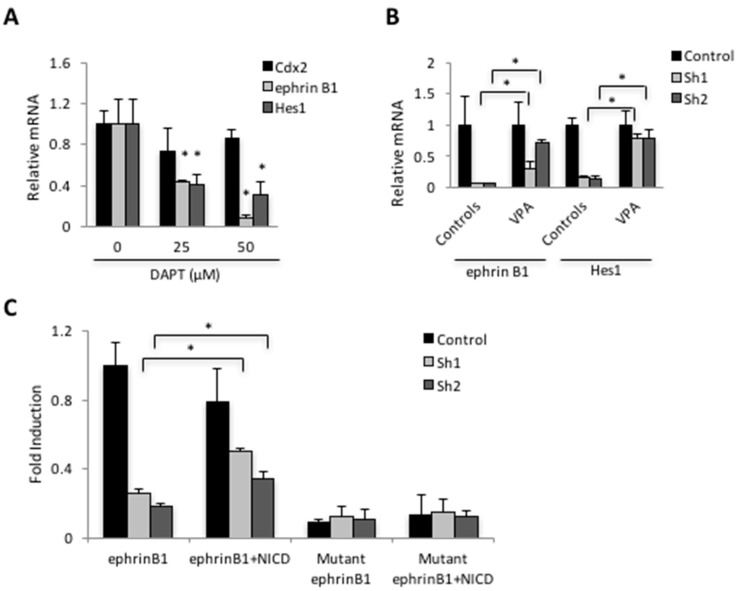
Notch signaling impacts the expression of *EphrinB1*. RT-PCR from control and SW480 Cdx2 knockdown cells treated with the Notch inhibitor DAPT (**A**) or with the Notch activator VPA (**B**). (**C**) Relative expression of a wild type or RBPJ mutant *EphrinB1* reporter in control and or Cdx2 knockdown SW480 cells with or without an *NICD* expression vector. Error bars represent the standard deviation from three independent samples. * *p* < 0.05 relative to control by Student’s *t*-test.

Cell-based reporter assays revealed that exogenous NICD was able to induce expression from sequences derived from the *EphrinB1* promoter, and that this response was lost upon mutation of the distal RBPJ binding motif in a manner comparable to mutation of the RBPJ motif in the *Hes1*-based reporter (Figure 3C). A role for Notch in activating *EphrinB1* was also supported by the observation that treatment with DAPT attenuated *EphrinB1* expression in SW480 cells in a manner comparable to that of *Hes1* (Figure 4A). Conversely, valproic acid (VPA), which has been shown to induce Notch signaling [37], caused an increase in the expression of both *EphrinB1* and *Hes1* in SW480 *Cdx2* knockdown cell lines (Figure 4B). The lack of a comparable gain in expression of *EphrinB1* and *Hes1* in the control cells treated with VPA may be indicative of rate-limiting VPA-sensitive Notch signaling in this cell line.

Taken together, the above findings suggest that Cdx2 impacts Notch signaling upstream of NICD. Consistent with this, reintroduction of the NICD into *Cdx2* knockdown SW480 cells induced expression from the *EphrinB1* reporter. This response was lost following mutation of the RBPJ binding site, irrespective of Cdx2 status (Figure 4C). Again, the lack of NICD-dependent regulation in wild type cells suggests a rate limiting event.

### 3.4. Loss of Cdx2 Enhances Stem Cell Character

**Figure 5:**
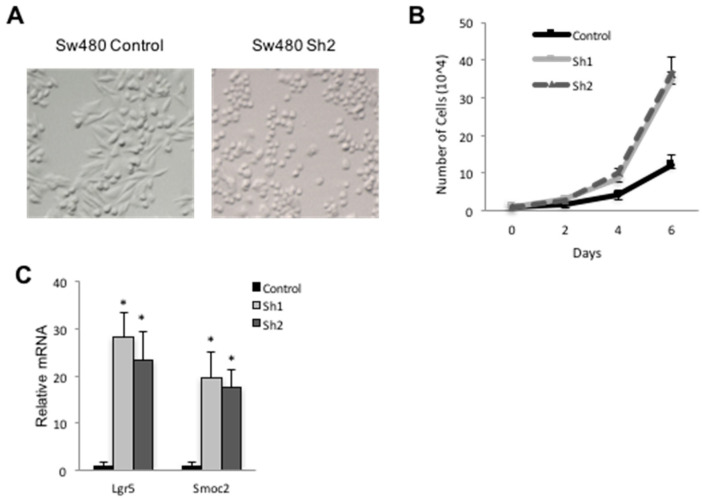
*Cdx2* knockdown increased expression of intestinal stem cell markers. (**A**) SW480 cells from control and *Cdx2* Sh knockdown cells. Note the lack of elongated cells in the knockdown culture. (**B**) Growth curve for control and *Cdx2* Sh knockdown cells. (**C**) RT-PCR from for stem cell markers in control and *Cdx2* knockdown SW480 cells. Error bars represent standard deviation from the mean expression levels of three independent samples. * *p* < 0.05 by Student’s *t*-test.

Both Cdx2 and Notch can serve to suppress CRC [5,38], and their loss of expression is associated with higher grade carcinomas in some cases [6,39]. SW480 cells are heterogenous and are composed of two populations of cells: cuboidal shaped epithelial cells (e-cells) and round shaped cells (r-cells) (Figure 5A). R-cells grow faster, produce larger colonies in soft agar and develop into larger, less differentiated tumors in nude mice [40]. We found that *Cdx2* knockdown in SW480 cells led to an increase in the proportion of r-cells, while control cells maintained predominantly an e-cell morphology (Figure 5A). *Cdx2* knockdown lines also grew at a faster rate (Figure 5B), an outcome also observed in CRC cells deficient in Notch signaling [41].

**Figure 6:**
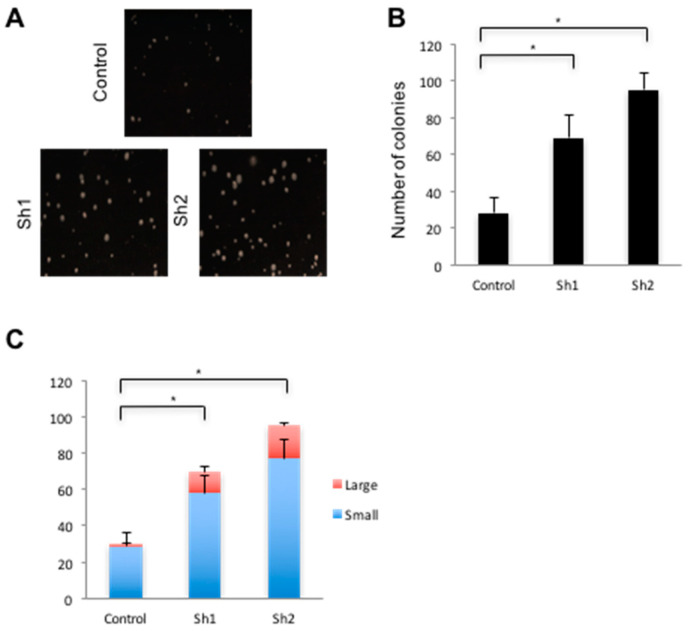
*Cdx2* knockdown leads to increased anchorage-independent growth. (**A**) Colonies grown in soft agar from control and *Cdx2* Sh knockdowns SW480 cells. (**B**) Quantification of number of colonies in soft agar and (**C**) Size distribution of SW480 colonies formed in soft agar. Error bars represent the standard deviation of five fields of view from three independent samples. * *p* < 0.05 by Student’s *t*-test.

Intestinal stem cells, or early progeny thereof, are thought to be the cells of origin in CRC [42]. Gene expression analyses revealed an increase in the intestinal stem cell markers *Lrg5* and *Smoc2* in lines lacking Cdx2 (Figure 5C), suggesting that cells deficient in Cdx2 exhibit a more stem-like character. Finally, *Cdx2* knockdown SW480 cells formed typical retractile colonies in soft agar, while wild type SW480 cells formed smaller colonies (Figure 6A). The *Cdx2* knockdown cells also formed significantly more (Figure 6B) and larger (Figure 6C) colonies relative to controls, consistent with a tumor-suppressive function of Cdx2 in this cell line.

## 4. Discussion

**Figure 7:**
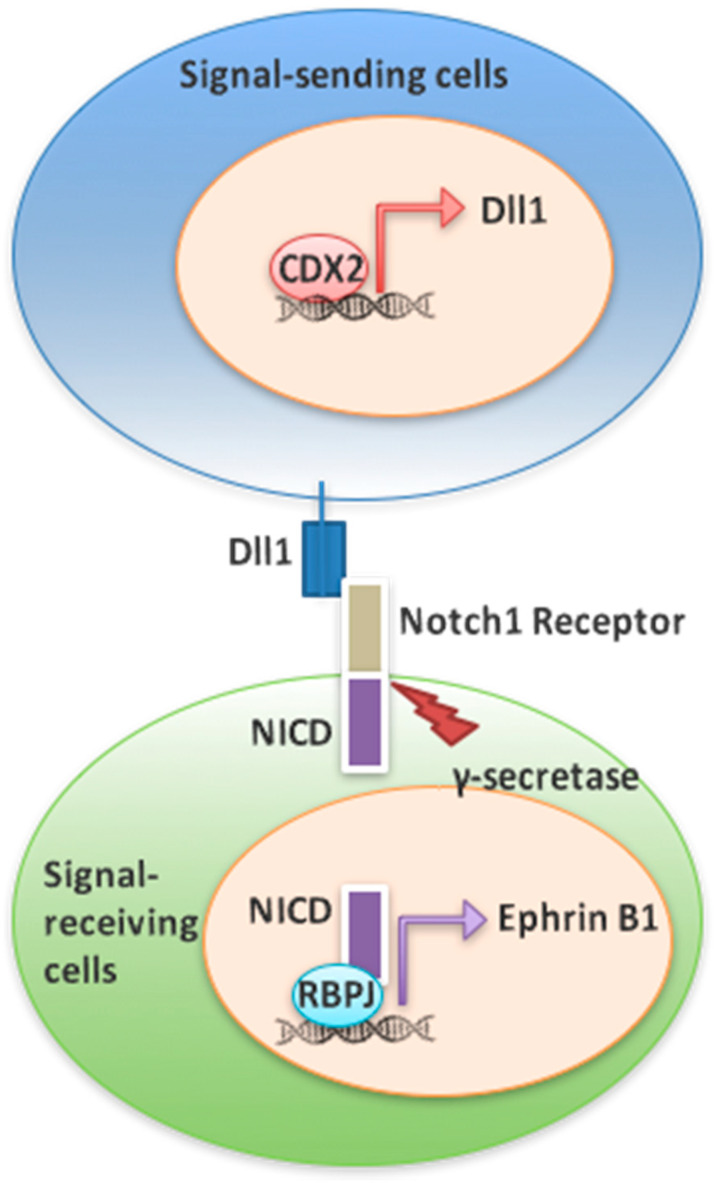
Model for Cdx2-dependent regulation of impact of *EphrinB1* expression. Cdx2 binds to the promoter of *Dll1* to effect Notch activation on an adjacent cell. Activation of the Notch receptor leads to cleavage of the NICD by γ-secretase and transcription of *EphrinB1*.

Although Cdx2 has known roles in intestinal homeostasis and exhibits tumor suppressive functions in some contexts [10,13,14,15,17], the mechanism by which it impacts these processes is largely unknown. To investigate this further, we developed SW480 cell lines deficient in Cdx2. This approach was necessary as these cells were refractory to CRISPR-Cas9 gene editing at this locus (our unpublished observation), consistent with prior work [21]. We found that loss of Cdx2 in SW480 cells led to a reduction in the expression of the target gene *Dll1* and attenuated Notch signaling. We further found that Notch is a direct regulator of *EphrinB1*, suggesting a mechanistic basis for the loss of *EphrinB1* expression and cell sorting defects previously described in *APC^min^-Cdx* compound mutants [19]. Finally, loss of Cdx2 increased the indices of stem cell character and promoted anchorage independent growth, again consistent with a tumor suppressive role for Cdx2 in CRC [10]. Taken together, our observations lead to a model wherein Cdx impacts *EphrinB1* expression and the CRC phenotype via a Notch-dependent mechanism (Figure 7).

Homeostasis of the intestinal epithelium requires coordinated interaction between numerous signaling pathways and transcription factors [43]. Cdx2 has been linked to regulation of expression of genes in the Notch signaling pathway [44], including *Dll1* [13,21]. Consistent with this, loss of Cdx2 in SW480 cells led to attenuation of *Dll1* expression concomitant with a reduction in Notch signaling, evidenced by diminished NICD levels and Notch reporter expression. Attenuation of Notch function was also evidenced by the increased expression of the secretory cell marker *Math1* in *Cdx2* knockdown cell lines, an outcome that is also seen following *Dll1* loss in the intestinal epithelium [45].

Notch function has been associated with expression of certain members of the Eph-Ephrin pathway in the intestine. For example, NICD can bind to, and regulate, an enhancer region in the *EPHB2* locus [46]. In human CRC cells, lesions in Notch signaling impair *EPHB3* enhancer function, while activation of Notch can induce *EPHB3* expression concomitant with a tumor suppressive response [41]. In agreement with prior observations [19], we found that Cdx2 impacts *EphrinB1* expression indirectly. Subsequent analysis revealed that *EphrinB1* is regulated directly by Notch, evoking a novel pathway by which Cdx2 regulates *EphrinB1* expression secondary to direct effects on Notch signaling pathway activity.

In the present study, we found that loss of Cdx2 in SW480 cells increased their proliferation and transformed nature, as well as increased expression of stem cell markers. This is in agreement with prior observations wherein attenuation of Cdx2 in colorectal cancer cells was shown to decrease expression of markers of differentiation concomitant with an increase in proliferation [12,47,48]. Conversely, Cdx2 overexpression has been shown to decrease mobility and tumor cell growth in vivo [49].

Cdx2 has both tumor promoter [50,51,52,53] and tumor suppressive potential [5,47,54,55]. Similarly, Notch activity is critical for adenoma formation in mouse models, but can also impair tumor progression [31,38,39,56]. Our results extend these observations and suggest that these effects of Cdx2 on tumor phenotype are mediated through Notch function and downstream targets such as *EphrinB1*.

## Data Availability

The data presented in this study are available on request from the corresponding author. The data are not publically available due to the lack of an appropriate repository for the nature of the experiments presented in this work.
